# Replication timing analysis in polyploid cells reveals Rif1 uses multiple mechanisms to promote underreplication in Drosophila

**DOI:** 10.1093/genetics/iyab147

**Published:** 2021-09-13

**Authors:** Souradip Das, Madison Caballero, Tatyana Kolesnikova, Igor Zhimulev, Amnon Koren, Jared Nordman

**Affiliations:** 1 Department of Biological Sciences, Vanderbilt University, Nashville, TN 37232, USA; 2 Department of Molecular Biology and Genetics, Cornell University, Ithaca, NY 14853, USA; 3 Institute of Molecular and Cellular Biology, Siberian Branch of Russian Academy of Sciences, 630090 Novosibirsk, Russia; 4 Laboratory of Structural, Functional and Comparative Genomics, Novosibirsk State University, 630090 Novosibirsk, Russia

**Keywords:** DNA replication, common fragile sites, replication timing, Drosophila, genome stability

## Abstract

Regulation of DNA replication and copy number is necessary to promote genome stability and maintain cell and tissue function. DNA replication is regulated temporally in a process known as replication timing (RT). Rap1-interacting factor 1 (Rif1) is a key regulator of RT and has a critical function in copy number control in polyploid cells. Previously, we demonstrated that Rif1 functions with SUUR to inhibit replication fork progression and promote underreplication (UR) of specific genomic regions. How Rif1-dependent control of RT factors into its ability to promote UR is unknown. By applying a computational approach to measure RT in Drosophila polyploid cells, we show that SUUR and Rif1 have differential roles in controlling UR and RT. Our findings reveal that Rif1 acts to promote late replication, which is necessary for SUUR-dependent underreplication. Our work provides new insight into the process of UR and its links to RT.

## Teaser

In polyploid cells, copy number is not uniform throughout the genome. Underreplication causes pericentric heterochromatin and defined regions of euchromatin to have reduced copy number relative to overall ploidy. SUUR and Rif1 are key regulators of underreplication, and Rif1 is a known regulator of replication timing (RT). Das *et al*. take a computational approach to measure replication timing in polyploid cells. Their results demonstrate that, while Rif1 and SUUR both promote underreplication, they differentially affect RT.

## Introduction 

Replication of the genome is a highly regulated process that requires duplicating billions of bases of DNA with a high degree of accuracy. Failure to properly replicate genetic and epigenetic information each and every cell cycle can result in cell lethality or disease (Jackson *et al.* 2014). Regulation of DNA replication occurs largely at the initiation stage, where in late M and G1 phases of the cell cycle, the origin recognition complex (ORC) facilitates loading of the MCM2-7 replicative helicase in an inactive state at all potential initiation sites (Bell and Labib 2016). In S phase, a subset of these helicase complexes will be activated by Dbf4-dependent kinase (DDK). S-CDK activity then facilitates replisome assembly and the formation of bidirectional replication forks emanating from the replication start site (Siddiqui *et al.* 2013). To ensure genome stability, not all helicases are activated simultaneously during S phase (Mantiero *et al.* 2011; Collart *et al.* 2013). Rather, helicase activation is regulated temporally in a process known as replication timing (RT) (Gilbert 2002; Rhind and Gilbert 2013). RT refers to the precise time in S-phase when a given genomic region gets duplicated. RT is correlated with chromatin structure and activity: regions of the genome that replicate early tend to be accessible and transcriptionally active, whereas regions that replicate late tend to be less accessible and less transcriptionally active (Gilbert 2002; Rhind and Gilbert 2013).

RT is not merely a passive reflection of the chromatin state, but rather an actively regulated process. One major regulator of RT is the trans-acting factor Rif1 (Rap1-interacting factor 1). Rif1 controls genome-wide RT from yeast to humans (Cornacchia *et al.* 2012; Hayano *et al.* 2012; Yamazaki *et al.* 2012; Peace *et al.* 2014; Sreesankar *et al.* 2015; Seller and O’Farrell 2018; Armstrong *et al.* 2020). The prevalent model for how Rif1 controls the RT program is based upon its conserved protein phosphatase 1 (PP1)-interaction motif. In this model, Rif1 recruits PP1 to loaded MCMs to oppose DDK-mediated helicase activation and to promote late replication (Davé *et al.* 2014; Hiraga *et al.* 2014, 2017). How Rif1 targets specific genomic regions or helicase molecules is unknown.

Despite the tight regulation of the DNA replication and RT programs, cell-type-specific regulation is necessary to accommodate cell-type-specific needs throughout development. For example, many cells of developing organisms are polyploid, having multiple copies of the genome in a single cell (Edgar and Orr-Weaver 2001; Lilly and Duronio 2005; Zielke *et al.* 2013). The genomes of polypoid cells, however, are not always fully replicated (Spradling and Orr-Weaver 1987; Edgar and Orr-Weaver 2001; Nordman and Orr-Weaver 2012; Hua and Orr-Weaver 2017). In Drosophila, most of the pericentric heterochromatin (PH) and certain euchromatic regions are underreplicated (eUR) in polyploid cells. These underreplicated regions resemble chromosomal fragile sites found in mammals in that they lack replication origins, are late replicating, display tissue specificity and are associated with DNA damage (Andreyeva *et al.* 2008; Nordman *et al.* 2011, 2014; Sher *et al.* 2012; Yarosh and Spradling 2014). Underreplication is an actively regulated process, and in Drosophila the SUUR (Suppressor of Underreplication) protein is required to promote UR (Belyaeva *et al.* 1998). SUUR is a potent inhibitor of replication fork progression and it promotes UR by inhibiting fork progression within specific regions of the genome (Sher *et al.* 2012; Nordman *et al.* 2014). SUUR, however, is unable to inhibit fork progression or promote UR on its own. Recently, we have shown that SUUR associates with Rif1 and recruits Rif1 to replication forks and that UR is completely dependent on Rif1 (Munden *et al.* 2018). Based on these data, we proposed that Rif1 acts downstream of SUUR to promote UR. Rif1 is known to regulate RT in multiple Drosophila tissues (Armstrong *et al.* 2020). It is unclear what contribution Rif1-dependent RT has on the promotion of UR, if any.

Here, we explore the relationship between UR and late replication and what role SUUR and Rif1 play in controlling UR and RT. To investigate this, we applied a sorting-independent computational method to profile RT in Drosophila polyploid cells genome wide, which has not been feasible with traditional methods. In addition to generating the first high resolution genome-wide RT profiles of salivary gland and fat body tissues, we discovered that SUUR and Rif1 have differential roles in controlling UR and RT. Whereas both SUUR and Rif1 are essential for UR, only Rif1 has a substantial effect on RT. Interestingly, our results also suggest that SUUR-mediated UR is dependent on late replication promoted by Rif1 in a chromatin-specific context. Together, our findings provide new insights into tissue-specific UR, its links to RT and the differential contribution of SUUR and Rif1 on RT and UR.

## Materials and methods

### Genomic DNA sequencing

Flies used in this study are listed in Table 1. Salivary glands were dissected in Ephrussi-Beadle Ringers (EBR) solution (10 mM HEPES pH 6.9, 130 mM NaCl, 5 mM KCl, 2 mM CaCl_2_) from ∼20 3rd instar female larvae prior to wandering per genotype, wild type—*OregonR*,* Rif1 - Rif^1^1/Rif^2^*, and *SuUR—SuUR^ES^*. Fat bodies were dissected in EBR from 25 OregonR female larvae per replicate 96 h after egg laying (AEL). Tissues were pelleted, resuspended in LB3 (10 mM Tris pH 8.0, 100 mM NaCl, 1 mM EDTA, 0.5 mM EGTA, 0.1% Na-deoxycholate, 0.5% N-Lauroylsarcosine), dounce homogenized and sonicated using a Bioruptor 300 (Diagenode) for 10 cycles of 30” on and 30” off at maximal power. Lysates were treated with RNase and Proteinase K and genomic DNA was isolated by phenol-chloroform extraction. Illumina libraries were prepared using NEBNext DNA Ultra II DNA Library Prep Kit for Illumina (New England Biolabs) following the manufacturer's protocol. Barcoded libraries were sequenced using Novaseq 6000 paired-end 150 bp sequencing.

**Table 1:** Strains used in this study

Reagent type (species) or resource	Designation	Source or reference	Additional information
Strain, strain background (*D. melanogaster*)	WT: *Oregon R*		
Strain, strain background (*D. melanogaster*)	*SuUR*	(Belyaeva *et al.* 1998)	*w^118^; SuUR^ES^*
Strain, strain background (*D. melanogaster*)	*Rif1^1^*	(Munden *et al.* 2018)	*w^118^*; *Rif1^1^*
Strain, strain background (*D. melanogaster)*	*Rif1^2^*	(Munden *et al.* 2018)	*w^118^*; *Rif1^2^*
Strain, strain background (*D. melanogaster*)	*Rif1*	(Munden *et al.* 2018)	*w^118^*; *Rif1^1^*/*Rif1^2^*

### Bioinformatics

#### RT generation

Bam files were aligned to dm6 (Dos Santos *et al.* 2015) by BWA mem (v0.7.17) (Li, 2013) and duplicate reads are marked with Picard Tools command “MarkDuplicates.” Coordinates of uniquely-mapping, nonduplicate, reads with a MAPQ >10 were extracted with samtools (Li *et al*. 2009) view (v1.11) (-F 1024 -F 256 -F 128 -q 10). All the salivary gland and fat body samples processed were in biological duplicates. RT values for wing disc and follicle cell were from previously published data (Armstrong *et al.* 2020).

RT was generated from a modified version of TIGER (Koren *et al.* 2021). Alignability filtering was performed against dm6 using a read length of 100 bp. The uniquely aligning sample reads were then partitioned into windows of 1000 uniquely alignable base pairs. GC correction was performed with standard segmentation (TIGER command “TIGER_segment_filt,” using the MATLAB function “segment”) parameters which temporarily removed eUR and PH regions for determining GC content bias. GC correction was then applied to all data (PH and eUR included).

From the GC-corrected data in 1 kbp windows, eUR regions were defined in WT fat body and salivary gland samples as regions of continuous low DNA copy number. In this, the raw DNA copy number data in 1 kb windows were segmented (TIGER command “TIGER_segment_filt,” R2 = 0.04, standard deviation threshold = 1) to identify regions of at least one standard deviation below the mean. From these regions, only those ≥50 kbp in length without gaps ≥10 kbp (≥10 continuous 1 kbp windows above the segmentation standard deviation cutoff) were called as eUR regions. This method provided the most accurate prediction of eUR regions by visual inspection. These predicted eUR regions overlapped with the smaller panel of previously published UR regions (Nordman *et al.* 2011) in fat body and salivary glands. The predicted and previously published eUR regions were merged with bedtools (Quinlan and Hall 2010) merge (v2.29.2) to finalize eUR zones in fat body and salivary gland.

Standard TIGER data filtering removes outlier windows attributed to noise or copy number variations. To still filter outliers, eUR and PH regions must be removed and filtered separately from the rest of the chromosome (arm regions). The eUR (as defined in the previous paragraph) and PH regions were removed from the GC-corrected data. In the arm-only regions, outliers were removed with segmentation (standard deviation threshold = 2, R2 = 0.06). eUR regions then separately filtered for outliers in a similar manner (standard deviation threshold = 3, R2 = 0.06). All segmentation parameters were chosen to optimize outlier removal via visual interpretation. The variable copy number of eUR and PH regions distorts normalization. Therefore, a second normalization was performed based only on the arm regions of chromosomes. The arm regions were first normalized to a mean of zero and a standard deviation of one. The mean shift and standard deviation values based on the arm regions were then applied to the eUR and PH regions.

The segmentation filtered arm, PH, and eUR regions were merged to form the final pre-smoothed RT (rawRT) data. These data were used for all the statistical analysis including variant calling between salivary gland and fat body samples. RT values were generated by smoothing the filtered data with a cubic smoothing spline (MatLab command “csaps,” smoothing parameter = 1 × 10^−^^15^). Only zones of >20 continuous 1 kbp windows were included and smoothing was not performed over gaps of >5 kpb. The smoothed profiles were then normalized to an individual chromosomal mean of zero and a standard deviation of one.

#### Quartile determination

To determine quartiles, we ranked the genomic windows according to their RT values. The top 25%, with the highest RT values, constituted the first quartile (Q1), representing the earliest replicating regions. Similarly, in a descending manner, the second (Q2) and third (Q3) quartiles contained the following 25% of genomic windows according to their RT values each. The fourth quartile (Q4) contained the genomic widows with the latest replicating regions.

#### Variant calling

The stats (v3.6.2) statistical package in R was used to identify 50-kb windows with significantly altered rawRT values, by one-way ANOVA test [aov, *P*-value adjusted for multiple testing with Bonferonni post-HOC correction {adjusted *P*-value < 0.01}]. Adjacent windows were merged and regions >200 kb in length were called as variant.

#### Correlation and autocorrelation

Correlation of RT profiles is generated from MATLAB command “corr” (values are Pearson’s r). Autocorrelation was calculated using the MATLAB command “autocorr” (number of lags = 1000).

#### RNA sequencing analysis

RNA-seq data for salivary gland was obtained from NCBI gene expression omnibus (GEO) (http://www.ncbi.nlm.nih.gov/geo/) under the reference series GSE31900. RNA-seq data for fat body were obtained from NCBI GEO (http://www.ncbi.nlm.nih.gov/geo/) under the reference series GSE25025. TopHat default parameters (v2.1.1) were used to align reads to the dm6 version of the Drosophila genome. Transcriptomes were generated using Cufflinks (v2.2.1). Transcript FPKM values for each RT window were generated by calculating the mean FPKM values for all the transcript regions overlapping the window. Overlap was determined by BEDTools intersect (v2.27.1) with −f 0.5 parameters.

#### ORC2 ChIP-seq analysis

ORC2 peak data for salivary gland was obtained from NCBI GEO (http://www.ncbi.nlm.nih.gov/geo/) under the reference series GSE31900. ORC2 peak data for fat body were obtained from NCBI GEO (http://www.ncbi.nlm.nih.gov/geo/) under the reference series GSE90916. Coordinates were converted to dm6 coordinates using the UCSC liftOver tool (Karolchik *et al.* 2004).

#### Miscellaneous bioinformatics

BEDTools intersect (v2.27.1) was used to determine overlap of RT windows with −f 0.5 parameters. Merging of adjacent RT windows was done by BEDTools merge (v2.27.1) with −d 11 parameters. Quartile cut-offs for each genotype or tissue were calculated by using the command “quantile, na.rm=T” of the stats (v3.6.2) statistical package in R. Coordinates from dm3 were converted to dm6 using the UCSC liftOver tool (Karolchik *et al.* 2004).

#### Indirect immunofluorescent staining

For immunostaining of polytene chromosome squashes, salivary glands from wandering third instar larvae were dissected in PBST (137 mM NaCl, 3 mM KCl, 8 mM NaH_2_PO_4_, and 2 mM KH_2_PO_4_; 0.1% Tween-20). Glands were then transferred into a formaldehyde-based fixative (0.1 M NaCl, 2 mM KCl, 10 mM NaH_2_PO_4_, 2% NP-40, 2% formaldehyde) for 1 min. Salivary glands were placed in an acetic acid–formaldehyde mix (45% acetic acid, 3.2% formaldehyde) for 1 min and squashed in 45% acetic acid. Squashes were snap-frozen in liquid nitrogen and coverslips were removed. Slides were incubated in 70% ethanol for 5 min twice and stored in 70% ethanol at −20°С. Slides were first washed three times in PBST for 5 min. Primary antibodies were added in a blocking solution (0.1% BSA in PBST) and incubated in humid chamber for 2 h at room temperature. The primary antibody dilutions used were as follows: rabbit polyclonal anti-SUUR (E-45) (Makunin *et al.* 2002), 1:50; mouse monoclonal anti-PCNA (PC10, Abcam, ab29) 1:500. Then, squashes were washed in PBST and incubated in secondary antibody (Alexa Fluor 488-conjugated goat anti-rabbit and Alexa Fluor 568-conjugated goat anti-mouse IgG antibodies, 1:500; Thermo Fisher Scientific) in blocking solution for 1 h. Squashes were mounted in VectaShield (Vector Laboratories) DAPI medium with 15 μg/mL DAPI. Images were acquired using an Olympus BX51 microscope equipped with a 100×/1.30 Uplan FI Ph3 oil objective and a DP70 camera.

## Results

### TIGER can be used to generate RT profiles from large polyploid cells

To understand the relationship between underreplication and RT on a genome-wide scale requires high-resolution RT and UR profiles. While current high-resolution UR profiles already exist, or can be generated by Illumina-based sequencing, methods to generate high-resolution RT profiles require FACS sorting of precise S-phase populations, which is not always technically feasible for large polyploid cells. To overcome this technical challenge, we have utilized TIGER (Timing Inferred from Genome Replication), a sequence-coverage based computational method that can measure RT without the need for sorting (Koren *et al.* 2021). Briefly, variations in DNA copy number driven by a modest percentage of cells in S phase within a population can be used to generate RT profiles. Therefore, RT profiles can be generated from Illumina sequence reads of a given cell- or tissue type if ∼10% or greater of the cells in the population are in S phase (Koren *et al.* 2014, 2021). TIGER has been used in mammalian cells to generate high-resolution genome-wide RT profiles and it rivals, or out performs, standard FACS-based methods to measure RT (Massey *et al.* 2019; Ding *et al.* 2020; Hulke *et al.* 2020).

**Figure 1:**
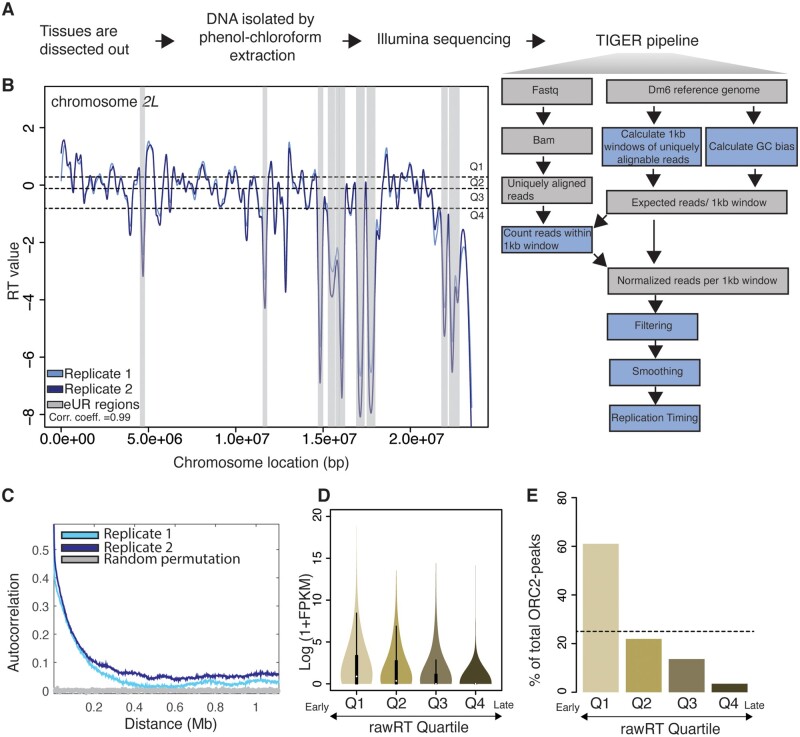
TIGER can be used to generate RT profiles from salivary gland polyploid cells. (A) Schematic outline of the workflow for preparing RT profiles through the TIGER pipeline: tissue dissection, DNA extraction, Illumina sequencing, and TIGER pipeline. (B) LOESS regression lines showing RT values for two biological replicates of wild-type salivary glands- replicate 1 (light blue) and replicate 2 (dark blue)- across the chromosome *2L* scaffold. See Supplementary Figure S1A for all other chromosome arms. Dotted lines mark the threshold of RT quartiles- Q1 (earliest) to Q4 (latest). Euchromatic Underreplicated regions (eUR) are highligted in gray. (C) Autocorrelation values plotted for two replicates of wild-type salivary gland- replicate 1 (light blue) and replicate 2 (dark blue). Gray line depicts autocorrelation values for random permutation of RT values. (D) RT windows from salivary gland were divided into quartiles with the highest rawRT values in Q1 and the lowest rawRT values in Q4. Average transcript FPKM values were calculated for every transcript within RT windows. The log2-transformed (1+FPKM) values were plotted for transcripts in each rawRT quartile in a violin plot. (E) Bar graph showing the percentage of ORC2-peaks corresponding to RT windows grouped into rawRT quartiles in salivary gland. The dotted line marks the average percentage of the ORC2-peaks when equally distributed across quartiles.

To determine if TIGER could be adapted for a polytene tissue, we dissected salivary glands from third-instar larvae, extracted genomic DNA and Illumina sequenced the genomic DNA (Figure 1A). We used 3rd instar larvae prior to the wandering stage to ensure that >10% of cells in the tissue were in S phase. The raw sequencing files were run through a TIGER pipeline adapted for Drosophila to calculate RT values and generate RT profiles (see *Materials and Methods*). As seen in Figure 1B, TIGER was able to generate profiles with characteristic peaks and troughs of a typical RT profile.

Several lines of evidence indicate the profiles generated by TIGER truly represent RT in the larval salivary gland. First, LOESS-smoothed profiles of biological replicates were nearly superimposable with a correlation coefficient (*r*) = 0.99 (Figure 1B, Supplementary Figure S1, A and B). Second, replicates show a high degree of autocorrelation, which indicates a high degree of spatial continuity typical of RT data (Figure 1C). Third, transcripts emanating from genes in early replicating regions have a higher abundance than transcripts emanating from genes in late replicating regions (Figure 1D) (Nordman *et al.* 2011). Fourth, early replicating regions are known to have a higher density of ORC2-binding sites (MacAlpine *et al.* 2010). To check if the relationship between ORC-distribution and RT holds true in our TIGER-generated salivary gland RT profiles, we divided the RT values in quartiles (Q1–Q4) where the first quartile had the earliest RT score and the fourth quartile had the latest RT score (see *Materials and Methods*). We used published ORC2 ChIP-seq data (Sher *et al.* 2012) and found that the percentage of ORC peaks is significantly higher in the earliest RT quartile Q1 (61.01%) compared to the latest RT quartile Q4 (3.47%) (Figure 1E). Finally, we compared patterns of replication defined cytologically by PCNA labeling in larval salivary gland polytene chromosomes to RT profiles generated by TIGER (Kolesnikova *et al.* 2018). Qualitatively, TIGER profiles had striking similarities to cytological patterns of PCNA labeling where we could confirm that late replicating, but fully replicated, cytological bands were also late replicating in our TIGER profiles (Supplementary Figure S1C). Quantitatively, on a larger scale, TIGER data recapitulates previously published measures of late replication with 78.4% of cytologically defined very late replicating regions on chromosome *2 L* falling within Q3 and Q4 of our TIGER measurements (Supplementary Figure S1D) (Kolesnikova *et al.* 2018). Taken together, the high autocorrelation patterning, positive correlation between early replication and high transcript abundance, positive correlation between early replication and ORC density and recapitulation of key RT differences by PCNA labeling of polytene chromosomes establish TIGER as an effective solution for sorting-independent RT profiling of a polyploid tissue.

One complication of measuring RT in polyploid cells by TIGER are the extreme differences in copy number within UR regions of the genome (Belyaeva *et al.* 1998; Nordman *et al.* 2011; Sher *et al.* 2012). Therefore, it might not be entirely possible to disentangle absolute RT values from copy number reductions within UR regions. We took two strategies to address this issue. First, to account for the variable copy number of UR regions, the RT values for the UR regions were normalized separately from the fully replicated regions of the genome. The generation of RT profiles by TIGER normalizes copy number to an average copy number of zero and a standard deviation of one (see *Materials and Methods*). However, due to the strong copy number disparity between replicated arm regions and the UR and PH regions, RT values were ultimately compressed. This posed an issue when comparing samples with and without UR and between cell types with different quantities of UR regions. Therefore, we isolated UR and PH regions and normalized the genome based on the average copy number and standard deviation within the consistent arm regions. Through this, we were able to normalize based on arm RT without the influence of variable UR or PH copy number. Second, rather than trying to provide an absolute RT value within UR regions, we divided the RT values in quartiles with the first quartile (Q1) representing the earliest replicating regions and the fourth quartile (Q4) representing the latest replicating regions. This allowed us to provide a relative RT value regardless of copy number differences within UR regions of the genome (see *Materials and Methods*).

### RT profiles generated with TIGER correlate with RT profiles generated by the G1/S method

**Figure 2:**
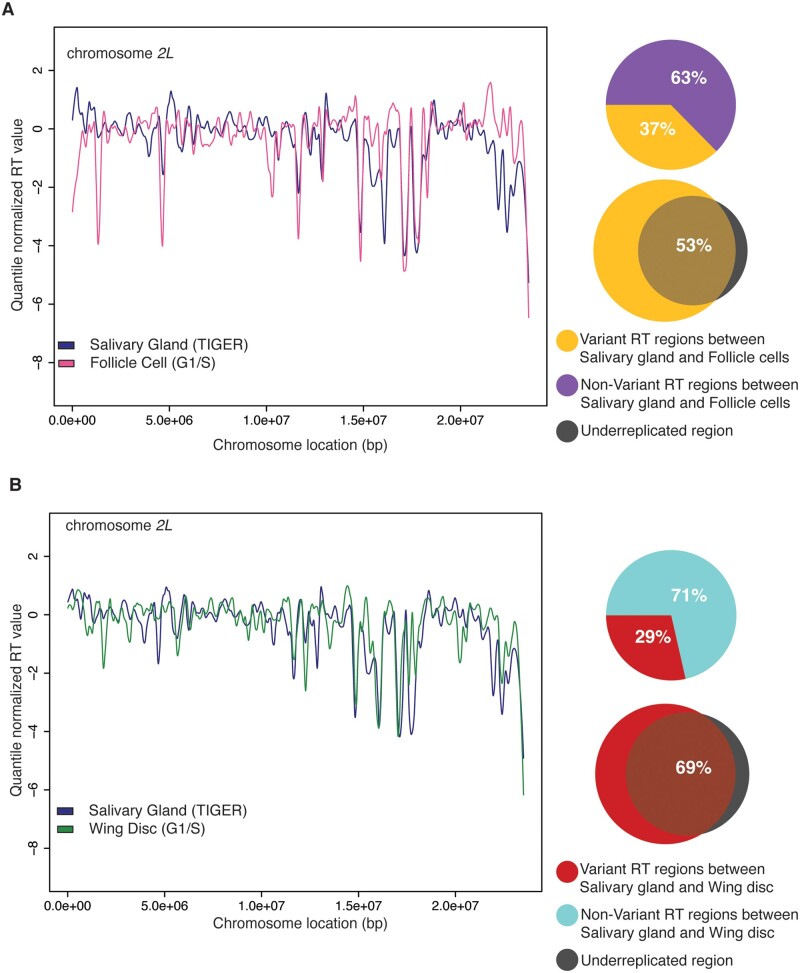
RT profiles generated with TIGER correlate with RT profiles generated by the G1/S method. (A) LOESS regression lines showing average wild-type salivary gland (blue) and wild-type follicle cell (brown)- replication timing values across the chromosome *2L* scaffold. See Supplementary Figure S2A for all other chromosome arms. Pie chart of all genomic windows of significantly different RT in follicle cell (yellow) and unchanged RT in follicle cell (purple) relative to the salivary gland across the major chromosome scaffolds. Venn diagrams comparing variant RT regions in the follicle cell (yellow) relative to salivary gland, and underreplicated region from both euchromatin and pericentromeric heterochromatin region (gray). (B) LOESS regression lines showing average wild-type salivary gland (blue) and wild-type wing disc (green)- RT values across the chromosome *2L* scaffold. See Supplementary Figure S2B for all other chromosome arms. Pie chart of all genomic windows of significantly different RT in wing disc (red) and unchanged RT in wing disc (light blue) relative to the salivary gland across the major chromosome scaffolds. Venn diagrams comparing variant RT regions in the wing disc (red) relative to salivary gland, and underreplicated region from both euchromatin and pericentromeric heterochromatin region (gray).

While the data presented in Figure 1 strongly suggests that the profiles generated by TIGER represent RT in the larval salivary gland, we wanted to compare these TIGER-generated profiles to RT profiles generated by conventional methods. Therefore, we compared directly TIGER-generated RT profiles in the larval salivary gland to RT profiles of larval wing discs and follicle cells of the adult ovary that our lab profiled using the G1/S method (Armstrong *et al.* 2020). If the TIGER-generated profiles truly reflect RT, then we would expect the RT profiles produced by TIGER to be comparable to these previously published data sets. Qualitatively, the RT profiles produced by both TIGER and G1/S methods exhibit similar nonrandom patterning (Figure 2, A and B, Supplementary Figure S2, A and B). Quantitatively we identified 63% of the genome in the follicle cells (Figure 2A) and 71% of the genome in the wing disc (Figure 2B) to exhibit similar RT relative to the salivary gland, compared by ANOVA (see *Materials and Methods*). While characterizing the variant RT regions, we noted that the majority of the variant RT regions in follicle cell and wing disc, compared to salivary gland, 53.38% and 68.52%, respectively (Figure 2, A and B), fall within known underreplicated regions of salivary glands. The differences in RT measured by TIGER and the G1/S method are consistent with expected differences due to cell-type specific changes in the RT program (Armstrong *et al.* 2020). Taken together, we conclude that TIGER is an effective method to generate genome-wide RT profiles from polyploid cells.

### Tissue-specific RT correlates with tissue-specific underreplication

Given that TIGER is an effective strategy to measure RT in polyploid tissues, it provides a tool to understand the relationship between tissue-specific RT and tissue-specific UR. Many larval tissues in Drosophila are polyploid and display tissue-specific underreplication (Nordman *et al.* 2011; Yarosh and Spradling 2014). For example, the larval fat body reaches a ploidy of ∼256C with defined tissue-specific underreplicated sites. Although it has been long known that late replication is associated with UR in polyploid cells, a quantitative exploration of the correlation between tissue-specific UR and RT was lacking. Therefore, we decided to profile larval fat body RT through TIGER to examine the relationship between RT and underreplication.

**Figure 3:**
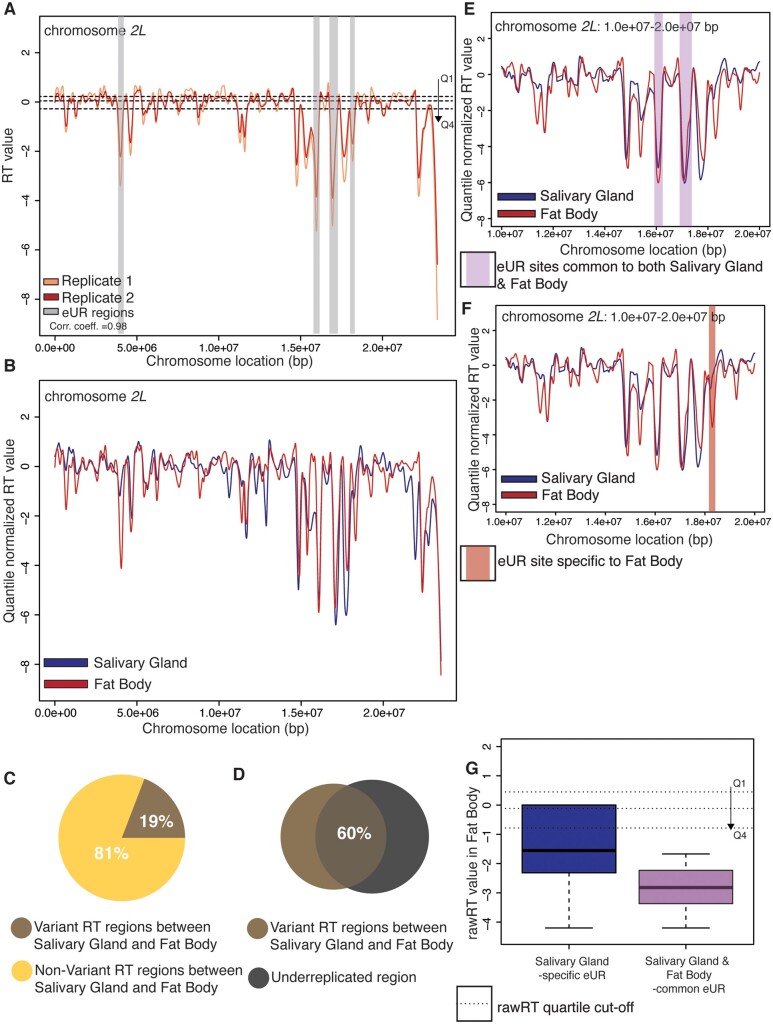
Tissue-specific RT correlates with tissue-specific underreplication. (A) LOESS regression lines showing RT values for two biological replicates of wild-type fat bodies- replicate 1 (light red) and replicate 2 (dark red)- across the chromosome *2L* scaffold. See Supplementary Figure S3A for all other chromosome arms. Dotted lines mark the threshold of RT quartiles- Q1 (earliest) to Q4 (latest). Euchromatic Underreplicated regions (eUR) are highligted in gray. (B) LOESS regression lines showing RT values for wild-type salivary gland (blue) and wild-type fat body (red)- across the chromosome *2L* scaffold. Each line represents the average of two biological replicates. See Supplementary Figure S3B for all other chromosome arms. (C) Pie chart of all genomic windows of significantly different RT in fat body (brown) and unchanged RT in fat body (yellow) relative to the salivary gland across the major chromosome scaffolds. (D) Venn diagrams comparing variant RT regions in the fat body (brown) relative to salivary gland, and underreplicated region from both euchromatin and pericentromeric heterochromatin region (gray). (E) LOESS regression lines showing RT values for wild-type salivary gland (blue) and wild-type fat body (red)- zoomed on two UR regions on chromosome *2L*, shared between salivary gland and fat body. The regions are highlighted with purple shading. (F) LOESS regression lines showing RT values for wild-type salivary gland (blue) and wild-type fat body (red)- zoomed on an UR region on chromosome *2L*, specific to fat body. The region is highlighted with red shading. (G) Box plot quantifying the distribution of rawRT values of the UR regions in fat body. UR regions are either salivary gland specific (blue) or shared between salivary gland and fat body (purple). Dotted lines represent cut-off values for the rawRT quartiles.

Larval fat body tissue was dissected from larvae 96 h AEL. We used this time point to ensure that at least 10% of the cells in the tissue were in S phase (Hua *et al.* 2018). Genomic DNA was extracted, Illumina sequenced and RT profiles were generated using TIGER as described (see *Materials and Methods*). Consistent with our observation in salivary gland, biological replicates were highly correlated (Figure 3A, Supplementary Figures S1A, S3, A and B; Corr. Coefficient = 0.98) and early replicating regions in fat bodies have higher transcript abundance and increased ORC2 peak density relative to late replicating regions (Supplementary Figure S3, D and E). Previously published RNA-seq and ORC2 ChIP-seq (Hua *et al.* 2018) data from fat body were used for these analyses.

Next, we quantified the variant RT regions between fat body and salivary glands and found that 19% of the genome showed RT differences between these two tissues (Figure 3, B and C, Supplementary Figure S3C). The majority of these variant regions (60.31%) fall within known underreplicated regions in salivary gland (Figure 3D). Notably, 44.51% of these variant regions still remain in the latest quartile of both the tissues. Importantly, we were able to identify tissue-specific RT variants that fall within underreplicated regions, raising the possibility that tissue-specific difference in RT could be correlated with tissue-specific UR.

Underreplicated sites that reside within the euchromatic regions of the genome (eUR sites) fall into two categories: those that are tissue-specific and those that are common between multiple tissues (Nordman *et al.* 2011). Given the tissue-specific nature of underreplication, we reasoned that it provides an opportunity to determine if tissue-specific changes in RT correlate with a tissue-specific UR. To this end, we compared the TIGER-generated RT profiles of larval salivary gland and fat body tissues, which are known to contain both tissue-specific and common sites of UR. If tissue-specific UR correlates with tissue-specific RT, then we would expect to see significant changes in RT at these loci. As expected, UR sites that are shared between both tissues are late replicating in both tissues (Figure 3E). We next focused on a site that is underreplicated uniquely in the fat body tissue. This fat body-specific UR site replicates earlier in salivary gland compared to fat body, suggesting that tissue-specific UR is correlated with tissue-specific RT (Figure 3F).

Since there were only a limited number of fat body-specific UR sites, we also performed the inverse analysis, and compared the RT status of Salivary Gland-specific UR sites in fat body. There are 40 UR sites present in Salivary Glands, of which 28 are specific to that tissue (Nordman *et al.* 2011). We took the genomic coordinates these 40 UR sites and segregated them into two categories- salivary gland-specific URs and URs shared between salivary gland and fat body. Next, we extracted the RT value for each of these categories for fat body. If tissue-specific underreplication is correlated with tissue-specific RT, we would expect to see a later RT value (in the fat body) for sites that are common between the two tissues then sites that are specific for the salivary gland. In agreement with this hypothesis, salivary gland-specific UR sites replicate earlier in the fat body when compared to UR sites that are shared between both tissues (Figure 3G). This establishes a correlation between late replication and tissue-specific underreplication.

### SUUR and Rif1 have differential effects on RT and UR

Loss of *SuUR* or *Rif1* function suppresses UR, however, the extent to which UR is suppressed in *SuUR and Rif1* mutants differs considerably. In an *SuUR* null mutant, UR within the euchromatin regions is nearly completely suppressed while UR in the PH is only partially suppressed (Belyaeva *et al.* 1998; Munden *et al.* 2018). In contrast, UR within euchromatin and pericentromeric heterochromatin appears to be fully suppressed in a *Rif1* null mutant (Munden *et al.* 2018; Kolesnikova *et al.* 2020). To ask if these differences in UR could be caused entirely by altered RT, we used TIGER to measure RT in *Rif1 and SuUR* null mutant larval salivary glands.

**Figure 4:**
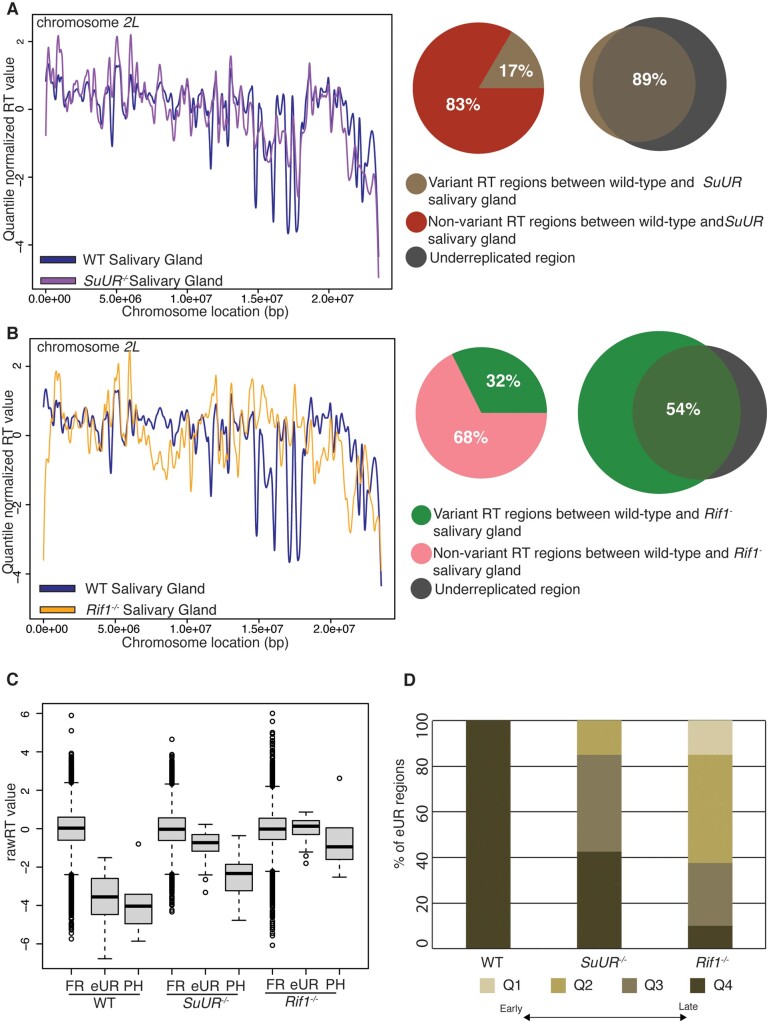
SUUR and Rif1 have differential effects on RT and UR. (A) LOESS regression lines showing average wild-type (blue) and *SuUR* mutant (purple) salivary gland RT values across the chromosome *2L* scaffold. See Supplementary Figure S4A for all other chromosome arms. Pie chart of all genomic windows of significantly different RT in an *SuUR* mutant (brown) and unchanged RT in an *SuUR* mutant (red) relative to wild-type across the major chromosome scaffolds. Venn diagrams comparing variant RT regions in *SuUR* mutant (brown) relative to wild-type and underreplicated region from both euchromatin and pericentromeric heterochromatin region (gray) in salivary gland. (B) LOESS regression lines showing average wild-type (blue) and *Rif1* mutant (yellow) salivary gland RT values across the chromosome *2L* scaffold. See Supplementary Figure S3B for all other chromosome arms. Pie chart of all genomic windows of significantly different RT in a *Rif1* mutant (green) and unchanged RT in a *Rif1* mutant (pink) compared to wild-type, across the major chromosome scaffolds. Venn diagrams comparing variant RT regions in *Rif1* mutant (green) relative to wild-type and underreplicated region from both euchromatin and pericentromeric heterochromatin region (gray) in salivary gland. (C) Box plot quantifying the distribution of rawRT values of the genomic regions that are fully replicated (FR), underreplicated and comes from euchromatic region (eUR) or underreplicated pericentromeric heterochromatin (PH) in wild type, *SuUR and Rif1* mutant salivary glands. (D) Bar-plot showing the distribution of rawRT values of eUR regions by rawRT quartiles, in wild type, *SuUR and Rif1* mutant salivary glands.

Genomic DNA was extracted from early third instar *SuUR* mutant salivary glands, sequenced and mapped reads were used to generate genome-wide RT profiles by TIGER. Similar to wild-type samples, biological replicates displayed high autocorrelation and high correlation between replicates, further supporting the robustness of TIGER (Figure 4A, Supplementary Figures S1A, S4, A and C). To quantitively measure the differences in RT between wild-type and *SuUR*, the genome was divided into nonoverlapping ∼50 kb windows and compared by ANOVA (see *Materials and Methods*). Comparison of RT profiles between wild-type and *SuUR* mutant salivary glands revealed that only 16.52% of the genome shows a significant difference in RT (Figure 4A). Given that RT profiles generated by TIGER rely on copy number measurements, we wanted to determine what fraction of RT changes reside within UR regions that are known to change copy number in an SUUR-dependent manner. 88.66% of the genomic regions that change RT in an SUUR-dependent manner fall within known UR regions in the *SuUR* mutant (Figure 4A).

Next, we used TIGER to generate RT profiles from *Rif1* null mutant larval salivary glands. Similar to wild-type and *SuUR* mutant data sets, biological replicates were highly correlated and displayed a high degree of autocorrelation (Figure 4B, Supplementary Figures S1A and S4D). Comparison of wild-type and *Rif1* mutant RT profiles of the larval salivary gland revealed that 32% of genomic regions had a significant change in RT (Figure 4B). Of all the regions that displayed differential RT in the *Rif1* mutant relative to wild type, only 53.38% fell within known UR regions (Figure 4B). This indicates that, in contrast to SUUR, Rif1 functions as a global regulator of RT in the polyploid larval salivary gland similar to its function in other diploid cell types (Cornacchia *et al.* 2012; Hayano *et al.* 2012; Yamazaki *et al.* 2012; Peace *et al.* 2014; Foti *et al.* 2016; Armstrong *et al.* 2020).

To determine the effect that SUUR and Rif1 have on RT, specifically within UR regions of the genome, we compared the RT values within UR regions. For this analysis, we separated UR regions that fall within the euchromatic arms of the genome (eUR) from the underreplicated PH that is mappable by short-read sequencing (PH). As expected, the RT values for the UR regions were significantly later than the fully replicated regions of the genome in wild-type salivary glands, as UR regions are known to be late replicating (Figure 4C) (Zhimulev *et al.* 1982, 2003; Nordman *et al.* 2011; Nordman and Orr-Weaver 2012; Nordman *et al.* 2014). In addition, the RT values within UR regions in wild-type salivary glands are likely artificially low due to extreme copy number differences in UR regions. Similar to wild type, eUR and PH regions of genome still displayed a pattern of late replication in the *SuUR* mutant (Figure 4C). In *SuUR* mutant, the difference in RT between the fully replicated and eUR or PH regions of the genome was not as substantial as in wild type. This is likely due to changes in copy number within UR regions, and not true changes in RT, as UR regions in the *SuUR* mutant are still some of the latest replicating regions of the genome (Figure 4A). In contrast to wild type and *SuUR* mutants, the eUR regions were not replicating later than the fully replicated regions of the genome in the *Rif1* mutant (Figure 4C). The PH in the *Rif1* mutant, however, replicates later than the fully replicated regions of the genome. To deconvolve the effect copy number has on the absolute RT values generated by TIGER, we compared distribution of RT values in eUR regions by RT quartiles for each genotype (Figure 4D). In wild type, all eUR regions fall within the latest replicating quartile. In the *SuUR* mutant 85% of the eUR regions remain in the two latest quartiles (Q3 and Q4). In contrast, in the *Rif1* mutant <40% of the eUR regions are found in the two latest quartiles. Strikingly, 15% of regions are found in the earliest replicating quartile (Q1). Together these data indicate two key points. First, changes in copy number due to UR can be separated from changes in RT, using TIGER. Second, SUUR and Rif1 have different effects on RT within UR regions. While eUR and PH are predominately late replicating in an *SuUR* mutant, only a subset of eUR regions and PH is late replicating in the *Rif1* mutant, compared to the fully replicated regions. Therefore, we conclude that loss of SUUR function suppresses UR independently of RT, whereas loss of Rif1 function results in both loss of late RT and loss of UR within eUR regions. This suggests that SUUR’s ability to promote UR at eUR regions depends on Rif1 to promote late replication within these regions.

### SUUR depends on Rif1 function to promote underreplication of eUR regions

**Figure 5:**
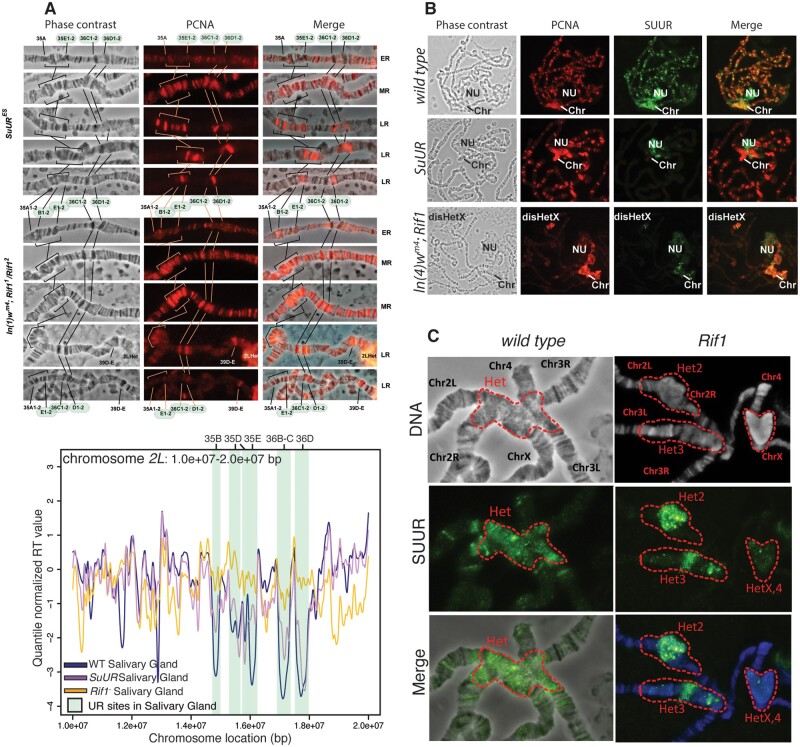
SUUR depends on Rif1 functions to UR of eUR regions (A) PCNA staining patterns in the 35A-36D region of *chr2L*. *35E*, *36C*, and *36D* are normally underreplicated. TIGER-generated RT profiles of the same region are shown for reference. ER, early replicating; MR, mid S phase replication; LR, late replicating. RT phases were determined by PCNA staining pattern in salivary glands. (B) anti-SUUR (green) and anti-PCNA (red) immunostaining of late replicating nuclei of the following genotypes: Oregon R (wt), *SuUR*, In(1)wm4; *Rif1*. Note that the *In(1)wm4* chromosome has an inversion that translocates a portion of heterochromatin on the *X* chromosome to *3C*. Chr, chromocenter; Chr3Het, Chromosome *3* heterochromatin; disHetX, distal heterochromatin of chromosome *X* transferred by inversion *In(1)wm4* to the region *3C*, NU, nucleolus. (C) anti-SUUR (green) immunostaining of late replicating nuclei of the following genotypes: Oregon R (wt), *Rif1*. Chr, chromosome; Het, heterochromatin.

TIGER-generated RT profiles for wild type, *SuUR and Rif1* mutant salivary glands make two key predications that can be tested cytologically. First, Rif1 mutants should have a replication pattern that is clearly distinct from wild type or *SuUR*-mutant salivary glands along the euchromatic arms. Second, *SuUR* binding to eUR sites (along the euchromatic arms of chromosomes) should largely be abolished since they are no longer late replicating in the *Rif1* mutant. SUUR binding to PH, however, should be largely unaffected in *Rif1* mutant polytene salivary glands. In support of the first prediction, the global pattern of EdU incorporation into chromosome arms of *Rif1* mutant salivary gland polytene chromosomes has recently been shown to differ significantly from that of wild-type or *SuUR* mutant polytene chromosomes. Sites that are normally late replicating and underreplicated in wild-type chromosomes appear to replicate earlier in S phase in a *Rif1* mutant (Kolesnikova *et al.* 2020). To extend this finding, we monitored the completion of replication of salivary glands by performing polytene squashes and staining chromosomes with anti-PCNA antibody to mark sites of active replication. PCNA labeling within the *35A* to *36D* region on chromosome *2L*, which contains several UR loci, was monitored for replication in different stages of S phase. In the *SuUR* mutant, UR regions at *35B*, *35E*, and *36D* all complete replication in late S phase, consistent with our TIGER measurements of RT (Figure 5A). In addition, the normally underreplicated region at *36C* completed replication prior to the underreplicated region at *36D* (Figure 5A). Consistent with this cytological observation, our TIGER RT profiles revealed that *36C* has an earlier RT value then *36D*. In contrast, these same regions have an earlier replication pattern in the *Rif1* mutant rather than late S phase in the *SuUR* mutant (Figure 5A). These specific RT differences validate the RT values we measured by TIGER. These cytological observations also validate our assertion that TIGER can measure RT even within the eUR regions.

Since UR is abolished in a *Rif1* mutant, and eUR regions replicate earlier in a *Rif1* mutant (Figure 4B and 4C), raised the possibility that SUUR requires Rif1 to associate with chromatin and/or replication forks in eUR regions. For example, SUUR is known to target late replicating regions of the genome, it should no longer bind to those regions if they replicate earlier in S phase. SUUR, however, should still bind to the late replicating PH in a *Rif1* mutant as SUUR was shown to localize to heterochromatin in a *Rif1* mutant and heterochromatin replicates late in *Rif1* mutant salivary glands (Munden *et al.* 2018). We monitored the localization of SUUR in wild-type and *Rif1* mutant polytene chromosomes using a SUUR antibody. As predicted, SUUR does not bind to the eUR regions in *Rif1* mutant salivary glands (Figure 5B). In contrast, SUUR still localized to the PH in *Rif1* mutant salivary gland chromosomes (Figure 5, B and C). We did observe, however, that the pattern of SUUR localization to the chromocenters was slightly altered in a *Rif1* mutant (Figure 5C). SUUR was not evenly distributed throughout the entire chromocenter, rather some regions of the chromocenter were stained more brightly than others and SUUR was often clustered in puncta (Figure 5C). Together, these cytological data complement our TIGER-generated RT profiles and indicate that SUUR requires Rif1 to associate with replication forks within these late replicating regions of the genome.

## Discussion

To understand the relationship between UR and RT, we have applied TIGER, a sorting-independent computational method to profile RT genome wide, to successfully measure RT in several polyploid tissues. By comparing genome-wide RT profiles between wild type, *SuUR and Rif1* mutants, we were able to further our understanding of the mechanisms Rif1 employs to promote underreplication (UR). We have found that SUUR and Rif1 have differential effect on RT. Furthermore, Rif1-dependent control of RT is critical for SUUR to promote UR in specific genomic regions. Our work has further emphasized the link between late replication and UR by demonstrating that tissue-specific UR is correlated with tissue-specific RT.

Methods to measure the genome-wide patterns of RT are dependent on FACS to isolate S and G1 phase populations (Ryba *et al.* 2011; Armstrong *et al.* 2018; Marchal *et al.* 2018; Hulke *et al.* 2020). FACS of large polyploid cells for RT profiling, however, is not efficient and this limitation has prevented profiling polyploid cells of the larval salivary gland, which has served as an important model for genome biology. Through the utilization of TIGER, we have overcome this challenge. This sorting-independent protocol is the latest addition to the repertoire of RT profiling methods and has proven to be both time saving and cost effective. We were able to generate TIGER-based RT profiles of multiple polyploid cells and several lines of evidence indicate that these profiles reflect true genome-wide RT profiles. RT profiles are highly reproducible between biological replicates, have a high degree of autocorrelation as a function of chromosome position, are correlated with transcript abundance and frequency of ORC2 binding sites and are visually similar to RT profiles of diploid tissues generated by the G1/S method.

TIGER-generated RT profiling of larval salivary glands revealed that while SUUR and Rif1 are both necessary for UR, they have significantly different effects on RT. While SUUR is responsible for a significant portion of UR (Makunin *et al.* 2002; Nordman and Orr-Weaver 2015; Munden *et al.* 2018), it has only a modest effect on RT. In contrast, Rif1 has a significant effect on RT. The fraction of the salivary gland genome that is dependent on Rif1 for RT is significantly greater than what was observed for Drosophila follicle cells or wing discs (Armstrong *et al.* 2020). While differences in the statistical methods used to perform variant calling could be responsible for subset of these differences, it is clear that Rif1 differentially affects RT in these three cell types. This is consistent with our previous observations showing that Rif1 controls RT in a cell-type-specific manner (Armstrong *et al.* 2020). It is still unknown, however, how Rif1’s activity is regulated to establish cell-type-specific patterns of RT.

**Figure 6:**
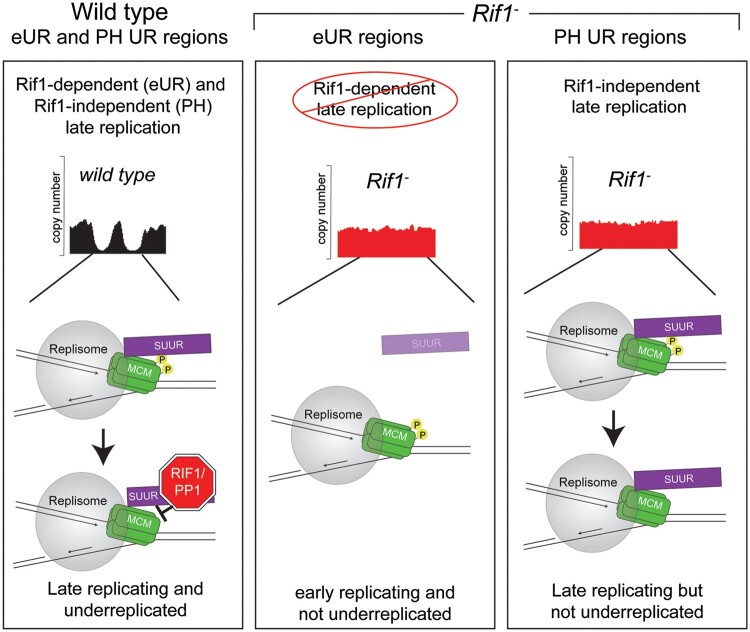
A model for Rif1-dependent promotion of underreplication. In this revised model, Rif1 first promotes late replication of certain genomic regions. Next, SUUR promotes underreplication in a subset of those late-replicating regions by recruiting Rif1/PP1 to replication forks to inhibit fork progression. Without Rif1, eUR regions replicate early and SUUR is unable to target replication forks in those regions. PH regions, however, remain late replicating in the absence of Rif1. Therefore, SUUR is able to target replication forks within PH regions. Without Rif1, SUUR cannot inhibit fork progression to promote UR.

Our previous work revealed that SUUR recruits Rif1 to replication forks to inhibit fork progression (Munden *et al.* 2018). RT profiling and SUUR localization in *Rif1* mutant salivary glands indicates that SUUR is dependent on Rif1 to promote late replication for SUUR-mediated UR. Thus, we propose a revised model for how Rif1 mediates UR (Figure 6). First, Rif1 promotes late replication of specific eUR and PH regions. Subsequently, SUUR localizes to these late replicating regions to inhibit replication fork progression in conjunction with Rif1. In the absence of Rif1, however, eUR regions that are normally underreplicated lose their late RT status and SUUR is unable to target replication forks within these regions. The PH UR sites, however, are still late replicating in absence of Rif1 likely due to additional factors that are dependent on H3K9 methylation status (Armstrong *et al.* 2019). SUUR still localizes to these PH regions, but is unable to inhibit fork progression and promote UR because Rif1 is not present and SUUR is unable to inhibit fork progression in the absence of Rif1 (Munden *et al.* 2018). This model is in agreement with multiple observations indicating that SUUR binding to euchromatic and PH is different and that the genetic requirements for UR in euchromatin and PH are unique (Guarner *et al.* 2017; Armstrong *et al.* 2019).

It is still unknown how exactly SUUR targets specific late-replicating regions of the genome to promote UR. In addition, it is unclear what aspects of chromatin structure and/or function change during development to generate tissue-specific patterns of UR. Data presented here demonstrate that tissue-specific RT correlates with tissue-specific UR. While late replication appears to be necessary for UR, it is not sufficient. Therefore, in addition to RT, there must be additional factors that help SUUR target specific genomic regions to promote UR.

## Data availability

All unique research materials and data will be freely available to other investigators. Data sets described in this manuscript can be found under the GEO accession number: GSE172375. Supplementary material included at figshare: https://doi.org/10.25386/genetics.15135105.
